# The Pattern of R2 Retrotransposon Activity in Natural Populations of *Drosophila simulans* Reflects the Dynamic Nature of the rDNA Locus

**DOI:** 10.1371/journal.pgen.1000386

**Published:** 2009-02-20

**Authors:** Jun Zhou, Thomas H. Eickbush

**Affiliations:** Department of Biology, University of Rochester, Rochester, New York, United States of America; Stanford University, United States of America

## Abstract

The pattern and frequency of insertions that enable transposable elements to remain active in a population are poorly understood. The retrotransposable element R2 exclusively inserts into the 28S rRNA genes where it establishes long-term, stable relationships with its animal hosts. Previous studies with laboratory stocks of *Drosophila simulans* have suggested that control over R2 retrotransposition resides within the rDNA loci. In this report, we sampled 180 rDNA loci of animals collected from two natural populations of *D. simulans*. The two populations were found to have similar patterns of R2 activity. About half of the rDNA loci supported no or very low levels of R2 transcripts with no evidence of R2 retrotransposition. The remaining half of the rDNA loci had levels of R2 transcripts that varied in a continuous manner over almost a 100-fold range and did support new retrotransposition events. Structural analysis of the rDNA loci in 18 lines that spanned the range of R2 transcript levels in these populations revealed that R2 number and rDNA locus size varied 2-fold; however, R2 activity was not readily correlated with either of these parameters. Instead R2 activity was best correlated with the distribution of elements within the rDNA locus. Loci with no activity had larger contiguous blocks of rDNA units free of R2-insertions. These data suggest a model in which frequent recombination within the rDNA locus continually redistributes R2-inserted units resulting in changing levels of R2 activity within individual loci and persistent R2 activity within the population.

## Author Summary

Transposable elements are abundant selfish components of all eukaryotic genomes. Despite the elaborate mechanisms eukaryotes have evolved to control these elements, they continue to proliferate. Here, we study R2 retrotransposons, highly successful elements that only insert into a site within the 28S rRNA genes of animals. In two natural populations of *Drosophila*, we show that R2 activity is directly linked to the level of R2 transcripts within each fly. The level of R2 transcripts is in turn linked to properties of the tandemly arranged rRNA genes, also known as the nucleolar organizer or rDNA locus. R2 transcript levels appear to depend upon the distribution of R2-inserted units within the rDNA locus, rather than the number of R2 insertions or the total size of the locus. Our findings suggest that R2 transcripts and hence activity can be prevented only when a fly can identify one or more large regions of the rDNA locus free of R2-inserted units. Because recombinations continually change the number and position of rDNA units with R2 insertions, individual loci switch between R2 inactivity and activity, thus perpetuating the long-term survival of R2 elements.

## Introduction

The abundant transposable elements present in eukaryotes have over the course of evolution helped shape the size, structure and expression of their genomes. Control over the spread of transposable elements has been suggested to be a consequence of the harmful effects of their insertions [1],[2] and involves either the epigenetic regulation of heterochromatin formation to block transcription [3] or small RNA pathways to degrade their RNA transcripts [4]. In spite of these controls, in most populations new insertions occur continuously at low levels. In *Drosophila melanogaster* for example, nearly half of spontaneous mutations are caused by transposable element insertions [5], and the insertion locations of most transposable elements are polymorphic in a population [1]. Multiple attempts have been made to monitor the activity of specific transposable elements in *D. melanogaster* and *D. simulans* [6]–[11]. However, it remains uncertain as to whether transposable elements maintain themselves by the inability of the control mechanisms to prevent all events, or the loss of transposable element control in a fraction of the individuals in a population.

**Figure 1:**
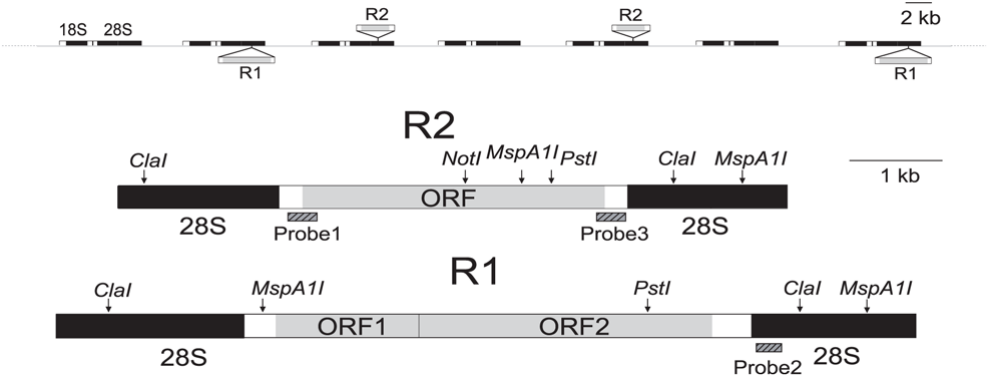
The rDNA locus of *D. simulans* and the site-specific retrotransposable elements, R1 and R2. The top panel shows the tandemly repeated rDNA transcription units with or without R1 and R2 insertions in the 28S rRNA gene. Every unit is composed of an 18S, 5.8S and 28S gene (black boxes) and transcribed spacer regions (white boxes). The R2 insertion site is located in the 28S rRNA gene 60 bp upstream of the R1 site. The bottom two diagrams show the R1 and R2 insertions in greater detail. The black boxes represent the 28S gene, the grey boxes represent the open reading frames of the R1 and R2 elements, and the white boxes represent the 5′ and 3′ untranslated regions of R1 and R2. *Cla*I, *Not*I, *Pst*I and *MspA1*I restriction enzyme cleavage sites used in this report, and the locations of the hybridization probes used for the RNA blots (probe1) and genomic DNA blots (probes 2 and 3) are indicated. *Not*I cleaves only the R2 elements but not R1 or the rDNA units.

The ribosomal RNA (rRNA) genes of eukaryotes are composed of hundreds to thousands of tandemly repeated units (the rDNA loci). Mature 18S, 5.8S and 28S rRNAs are processed from the single transcript of each rDNA unit. Frequent recombination (unequal crossovers) within these rDNA loci removes sequence variation, a process referred to as concerted evolution [12]. Concerted evolution is so efficient almost no nucleotide sequence variation exists between the different rDNA units of a locus [13],[14]. Given this ability of the locus to rid itself of variation, it is surprising that the rDNA locus is home to many specialized transposable elements [15]–[17]. For example, R1 and R2 are non-LTR retrotransposable elements that insert specifically into the 28S rRNA genes (Figure 1). R1 and R2 are highly adept at maintaining themselves in the rDNA locus. They are found in most arthropods and appear to have been vertically inherited since the origin of the phylum [18],[19]. Remarkably, R2 elements appear to have been inserting into the same site of the large subunit rRNA gene since near the origin of metazoans [17]. Within many insect species a large fraction of the rDNA units are inserted by R1 and R2 suggesting rapid rates of insertion [20]–[22]. Even though these many inserted units can not make functional 28S rRNA, the host does not appear unduly affected because in most organisms many more rDNA units are encoded than are needed for the production of rRNA [23].

An analysis of inbred laboratory stocks of *D. simulans* originally derived from a population in Paradise, CA revealed lines with no R2 activity as well as lines with extremely high rates of R2 retrotransposition [24],[25]. The R2 retrotransposition activity was found to be under transcriptional control with the genetic differences between active and inactive stocks closely linked to the rDNA locus [26]. Minor, if any, influence on the level of R2 activity was found associated with the autosomes. Analysis of the rDNA loci from the active and inactive stocks revealed that the numbers of R2 elements in the active stocks were on average twice that found in the inactive stocks. The R2 elements in the active stocks were distributed throughout the rDNA locus suggesting a model in which a high density of R2 elements prevents the host from activating the needed number of uninserted rDNA units without also activating R2-inserted units.

Because the active and inactive stocks used in these studies had been maintained in the laboratory for over 10 years, and considerable changes in the structure of the rDNA locus had occurred during that time [25], it was unclear whether R2 activity in these laboratory stocks were an accurate reflection of the levels and pattern of R2 activity in natural populations. Here we studied R2 element activity in two populations of *D. simulans* immediately after their capture. A 100-fold range of R2 transcript levels was detected and correlated with R2 retrotransposition activity. However, the number of R2 elements in the rDNA loci of the active and inactive lines did not substantially differ, suggesting the previously detected accumulation of R2 in active stocks was the result rather than the cause of R2 activity. The property of the rDNA locus that best correlated with R2 activity was subtle changes in the distribution of elements within the rDNA locus.

## Results

Our previous studies suggested that the levels of R2 transcription and retrotransposition in *D. simulans* are controlled by the composition of the rDNA locus located on the X chromosome [26]. Because the rDNA locus of stocks in which the R2 elements are active can change rapidly [25], the pattern of R2 activity in natural populations needed to be assayed within a few generations after the flies were captured. To this end, lines containing a single rDNA locus (iso-rDNA lines) were generated in two generations by crossing individual wild males to females with Beadex, a phenotypic marker near the rDNA locus (see Materials and Methods). In total, 88 iso-rDNA lines from flies collected in San Diego and 92 lines from flies collected in Atlanta were generated and used for the initial screening of R2 transcript levels.

### R2 Transcript Levels in Natural Populations

**Figure 2:**
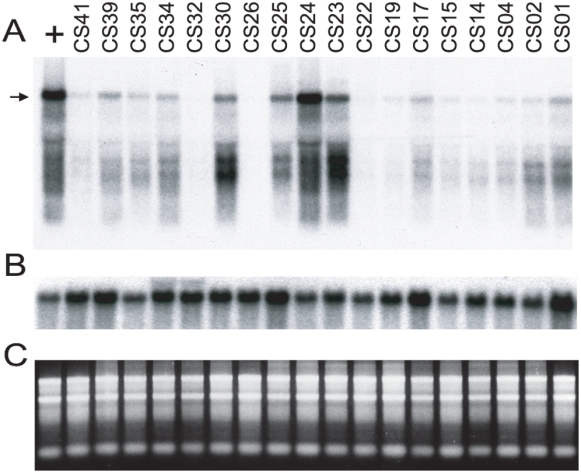
R2 transcripts levels in the iso-rDNA lines derived from the Atlanta and San Diego populations. (A) Representative RNA blot using RNA isolated from 18 iso-rDNA lines and, as a positive control (lane labeled, +), laboratory line 58, known to have high levels of R2 transcription and retrotranspositions [26]. The arrow indicates the 3,600 nt full-length R2 transcript. For each lane 10 µg of RNA isolated from adult females was fractionated on a 1% agarose gel, transferred to a nylon membrane, and probed with a 300 nt R2 5′ antisense RNA (probe1 in Figure 1). (B) As a control for RNA quality and loading the R2 hybridization signal was allowed to decay and the blot was re-probed with a fragment of the alcohol dehydrogenase gene [26]. (C) Ethidium bromide staining of the RNAs used for the analyses in panels A and B.

R2 transcript levels were monitored in RNA isolated from adult females the first generation after establishing the iso-rDNA lines. To enable comparisons between different blots, RNA from line 58, a laboratory stock previously shown to have stable high levels of R2 transcription and retrotransposition, was included in each analysis [25],[26]. A representative RNA blot is shown in Figure 2. Panel A is the RNA probed with R2 sequences, panel B is the same blot probed with a control gene (alcohol dehydrogenase), and panel C is the ethidium bromide staining pattern of the RNAs used. The 3,600 nt hybridizing band detected at various intensities in panel A represents full-length R2 element transcripts [26]. Because the hybridization probe used in the blot was from the 5′ end of the R2 element (Figure 1, probe 1), the series of lower hybridizing bands represent intermediate degradation products of full-length transcripts, rather than the transcripts from 5′ truncated R2 elements. These lower hybridizing bands between lines generally corresponded in relative intensity to the 3,600 nt band suggesting the lines had similar rates of R2 RNA degradation.

**Figure 3:**
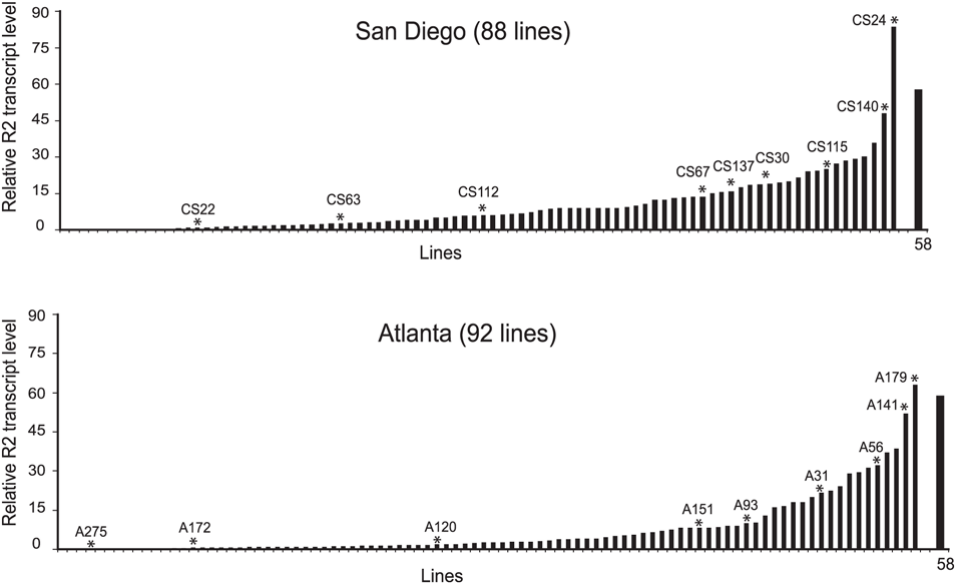
R2 transcript levels of 180 iso-rDNA lines derived from populations in San Diego and Atlanta. The 3,600 nt full-length R2 transcript detected on RNA blots of adult female RNA (see Figure 2) were quantified on Phosphorimager scan and ImageQuant software. To control for RNA loading and possible degradation the R2 signal was standardized to the alcohol dehydrogenase signal from the same blot. RNA from line58, an active laboratory stock, was also present on every RNA blot as a standard to enable comparisons between blots. The 18 lines selected for further analysis to monitor R2 retrotranspositions and to determine the compositions of their rDNA loci are indicated with asterisks and labeled.

Shown in Figure 3 is a summary of the R2 transcript levels in all 180 iso-rDNA lines. The populations from both Atlanta and San Diego had transcript levels that varied over a wide range. Both populations also had similar fractions of the lines at comparable transcript levels. About 45% of the stocks derived from the San Diego population and 60% of the stocks from the Atlanta population had no or extremely low levels of R2 transcripts (defined here as hybridization less than 5 times the background hybridization to the filters). The remaining lines from each population had R2 transcript levels that varied in a continuous manner from 6 to 85 times background. Only four lines (two from each population) had transcript levels similar to or greater than the R2 active laboratory stock, line 58.

To determine if the R2 transcript levels rapidly changed while being maintained in the laboratory, 32 lines that spanned the full range of R2 transcript levels from the two populations were re-assayed either 2 or 3 generations after the initial screening. No line changed significantly in transcript levels. Lines with intermediate levels of transcription showed the highest levels of change between the two generations with some lines increasing and others decreasing by 20% (data not shown).

### The Natural Lines Contained Independent rDNA Loci

Nine lines from each population were selected to represent the observed range of R2 transcript levels (lines indicated with asterisks in Figure 3). Because the flies for each population were collected from one location over a period of only a few days, some of the flies could be closely related and thus not represent independent rDNA loci. To determine the relatedness of the rDNA loci from these lines, the specific R2 insertions in each of the 18 lines were compared. R2 elements generate identical 3′ junctions with the 28S gene but about one-half of the copies have variable 5′ junctions [27]. This 5′ end variation predominantly corresponds to truncations and has been suggested to arise from the R2 polymerase failing to reach the 5′ end of the R2 transcript during reverse transcription and/or from DNA repair processes associated with partial retrotransposition events [15]. The 5′ ends of the R2 elements from each line were amplified in a series of PCR reactions, and the products separated at single nucleotide resolution on high-resolution sequencing gels. These R2 5′ truncation profiles serve as a robust means to determine whether the rDNA loci of the different lines are related. For example, laboratory stocks with inactive R2 elements continue to have identical 5′ truncations after 100 generations [24],[27], while 60–90% of the truncations are identical after 30 generations when the R2 elements are active [25].

**Figure 4:**
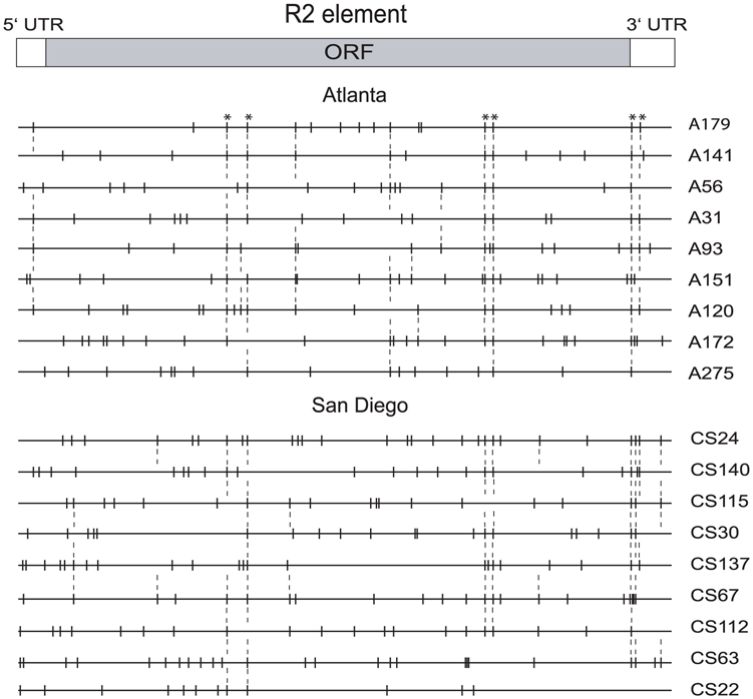
R2 5′ truncation profiles of 18 representative *D. simulans* lines. Diagramed at the top is a full-length R2 element with the location of the ORF and 5′ and 3′ untranslated regions indicated. Shown below the R2 diagram are the individual 5′ truncation profiles of each iso-rDNA line. Each horizontal line represents one of the *D. simulans* lines with the position of the vertical lines indicating the 5′ end point of a truncated R2 element in that line. Because all R2 insertions have an intact 3′ end of the element, each 5′ truncated R2 copy extends from the vertical line to the 3′ end of the R2 element. The collection of the 5′ truncated R2 copies differs for each line. Those 5′ truncations connected by dotted lines are shared between lines with those dotted lines indicated with an asterisk shared between the two populations. Full-length R2 elements in each line can also show small differences in length but are not shown. For each population the lines are arranged from the highest level of R2 transcript (top) to the lowest level of transcripts (see Figure 3).

Shown in Figure 4 are the collections of 5′ truncated R2 insertions found in the 18 lines. The distances from the vertical lines to the 3′ end of the element represent the lengths of the different 5′ truncated elements in each line. Each line had from 13 to 28 (mean 20) different length 5′ truncated copies. From 2 to 9 (mean 6) of these elements were shared with one or more other lines (identified in the figure with dotted vertical lines). These shared copies consisted largely of six specific truncations (vertical dotted lines marked at the top with asterisks) that were also shared between the Atlanta and San Diego populations. These long-term stable R2 copies are presumably located in regions of the rDNA locus, perhaps the edges, which seldom undergo recombination events. These data indicate that the rDNA loci in the 18 lines selected for further study are not closely related with their rDNA loci having undergone significant turnover of R2 elements since their common ancestor. Finally, the 5′ truncation profile of each line also differed from the rDNA loci present in the Bx line (not shown), indicating no instance of recombination between the Bx marker and the rDNA locus during the establishment of the iso-rDNA lines.

### Correlation of R2 Transcript Levels with Retrotransposition

R2 activity (retrotranspositions) was next scored in the 18 representative lines. To monitor new retrotransposition events, the R2 5′ truncation profiles of 16 males from each line in the eighth generation after formation of the iso-rDNA lines were compared to the profile of the original male used to establish each line. Retrotransposition (insertion) events were scored as additional R2 5′ truncations observed in any of the male progeny. Because R2 deletions have also been correlated with R2 retrotransposition activity [25], R2 copies present in the original rDNA loci but missing in the eighth generation loci were also scored as R2 activity. R2-induced deletions typically involve multiple rDNA units [25], thus all deletions within a loci were scored as single events, independent of the number of R2 copies involved. Previous analyses have suggested that the insertion and deletion of 5′ truncated copies of R2 are similar to full-length copies, thus these 5′ truncated events represent about half of the total retrotransposition events within the locus [10],[24].

**Figure 5:**
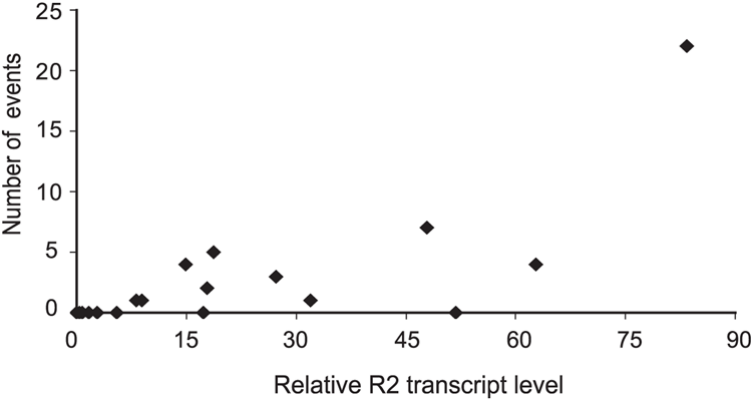
Correlation between R2 transcript levels and the rate of R2 retrotransposition. The 18 iso-rDNA lines described in Figures 3 and 4 were assayed for R2 retrotransposition activity after eight generations. The R2 5′ truncation profiles of 16 males from the eighth generation were compared to the R2 profile from the original male used to establish each line. Because R2 deletions are also associated with R2 activity [25] loss of pre-existing insertions were also classified as R2 events. Because multiple R2 elements can be eliminated by a single deletion, all losses of R2 copies were scored as single events regardless of the number of R2 elements involved. For each line the total number of R2 events in all eighth generation males was plotted versus the R2 transcript level of that line.

A summary of the retrotransposition activities of the 18 lines plotted versus their relative R2 transcript levels is shown in Figure 5. No changes in the 5′ truncation profiles were scored in the six low transcript lines. The 12 lines with readily detected levels of R2 transcripts had on average 4 events (range 0 to 22). For comparison our laboratory stocks with the highest levels of R2 retrotransposition typically generated 20–30 events in similar experiments [24],[25]. While not all lines with high levels of transcript had measurable levels of retrotransposition, R2 transcript levels were positively correlated with R2 retrotransposition (Spearman rank correlation test, r = 0.71, p = 0.0005). If the one line with the highest level of activity (CS24) was excluded from the analysis, there still remained a positive correlation (r = 0.67, p = 0.001). Re-examination of the high line (A141) in Figure 5 with no retrotranspositions revealed one retrotransposition event. The lower levels of retrotransposition in some lines with significant R2 transcript levels suggest there may be other levels of control over R2 activity besides transcription.

Because of the large number of R2 events that had accumulated in the most active line (CS24), it was possible to score retrotransposition events in this line at earlier generations. Six events were detected in the first generation after founding the iso-rDNA line, and nine events had accumulated by the third generation. Thus, as in our previous studies of inbred laboratory stocks, high retrotransposition activity can be stable over multiple generations.

### Correlation of R2 Transcript Levels with the Number of R2 Copies and rDNA Locus Size

The total number of R2 copies in each of the 18 lines was determined by directly counting their different 5′ ends. In addition to the 5′-truncated copies shown in Figure 4, about one-half of the R2 elements are full-length (i.e. extend to the first nucleotide of the consensus sequence). Many of these full-length copies can also be individually counted in PCR assays because they too exhibit length variations associated with small deletions of the 28S gene and non-templated nucleotide additions [28]. To estimate the copy number of those elements with identical length 5′ ends, the intensity of each PCR band was calculated with a regression analysis using single copy variants as reference markers [29]. The total number of R2 copies in the 18 lines varied from a low of 33 to a high of 69, while the number of full-length R2 elements varied from 18 to 34 (Table 1).

**Table 1:** R2 elements and locus composition in 18 *D. simulans* lines.

	R2			Locus			
	FL	5′ Trun	Total	% Un	% R1*	% R2*	Total
A179	21	18	39	80.0±2.0	4.4±0.2	20.0±1.8	195±18
A141	26	22	48	75.1±2.7	10.7±0.7	21.6±2.3	222±24
A56	34	27	61	73.0±3.1	10.3±0.4	23.6±2.7	259±30
A31	27	24	51	72.0±3.5	11.0±0.5	27.6±3.2	185±22
A93	22	23	45	73.2±3.0	10.9±0.5	24.3±2.6	186±20
A151	28	24	52	79.6±3.1	5.7±0.4	20.4±2.8	255±36
A120	25	23	48	77.2±2.5	11.7±1.0	16.7±1.7	287±30
A172	32	30	62	83.6±2.6	5.8±0.7	15.7±1.9	395±47
A275	18	19	37	83.8±2.7	5.8±0.7	16.1±2.1	230±31
CS24	27	32	59	77.5±2.7	7.9±0.5	22.5±2.3	262±27
CS140	25	32	57	69.9±2.7	11.7±0.6	26.0±2.1	219±18
CS115	29	23	52	73.4±2.8	9.1±0.8	25.5±1.9	204±15
CS30	25	22	47	75.4±1.4	10.2±0.6	22.0±1.2	214±12
CS137	31	38	69	69.9±3.0	13.3±1.4	22.7±2.0	304±27
CS67	26	25	51	75.2±1.5	8.7±0.1	23.4±1.4	218±13
CS22	21	12	33	82.1±1.4	8.3±0.4	16.4±1.2	201±15
CS63	18	33	51	83.0±1.6	6.8±0.5	15.8±1.3	323±27
CS112	25	20	45	78.9±1.8	6.5±0.3	20.7±1.7	218±18

FL, full-length R2 copies; 5′ Trun, 5′ truncated R2 copies; Un, uninserted rDNA units; R1, R1-inserted units; R2, R2-inserted units.

*: Values include both single and R1+R2 (double) inserted units.

**Figure 6:**
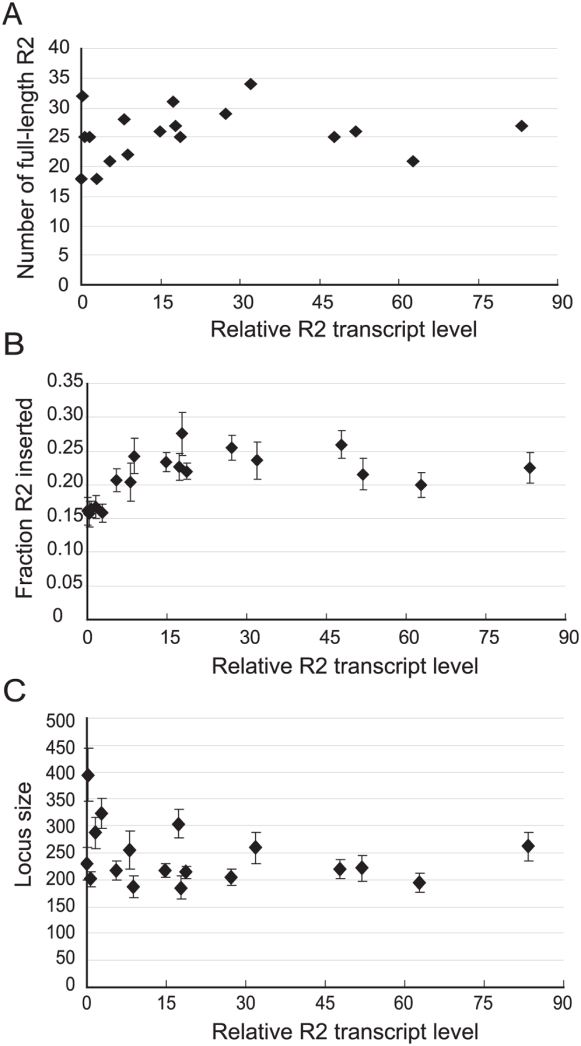
Relationship of R2 transcript levels to the size and composition of the rDNA locus. (A) The relative R2 transcript level of each line is plotted versus the number of full-length, thus potentially active, R2 elements in that line. (B) The level of R2 transcripts in each line is plotted versus the fraction of the rDNA units inserted with R2 elements. The fraction of the rDNA units inserted with R2 was determined using genomic blots (see Materials and Methods). (C) The level of R2 transcripts is plotted versus the size of the rDNA locus. The total number of units is determined by dividing the total number of R2 elements by the fraction of units inserted with R2 for each line.

Figure 6A plots the number of full-length and thus potentially active R2 copies versus the level of R2 transcription in each of the 18 lines. The six lines with low R2 transcript levels had on average 23.3 full-length copies, while the six lines with highest transcript levels had on average 26.8 copies. However, a Spearman rank correlation test suggested no correlation between R2 number and transcript level (r = 0.28, p = 0.19).

We next determined if R2 transcript levels correlated with the fraction of the rDNA units inserted with R2. Because the 3′ ends of all R1 and R2 elements are identical, blots of genomic DNA digested with select restriction enzymes place the uninserted rDNA units, the 3′ ends of R1-inserted units and the 3′ ends of R2-inserted units on different sized fragments. These blots when hybridized with a segment of the 28S rRNA gene immediately downstream of the R1 insertion site reveal the percentage of the total rDNA units that are of each class [30],[31]. To score those R2 copies that are inserted in the same rDNA unit as an R1 element, a second genomic digest was conducted which placed the 3′ ends of R2 elements in single and double inserted units on different sized restriction fragments and probed with the 3′ end of the R2 element (probe 3, Figure 1) [10],[24]. The fraction of the rDNA locus inserted with R2 elements (both full-length and 5′ truncated) varied from 16% to 28% (Table 1). Plotted in Figure 6B is the fraction of the rDNA units inserted with R2 versus the R2 transcript levels. About 17% of the rDNA units in the lowest R2 transcript lines were inserted with R2, while about 23% of the rDNA units were inserted in the highest transcript lines. While there is a positive correlation between transcript level and fraction inserted when all lines are included (r = 0.61, p = 0.003), there was no correlation when the six low transcript lines were removed (r = −0.17, p = 0.30).

**Figure 7:**
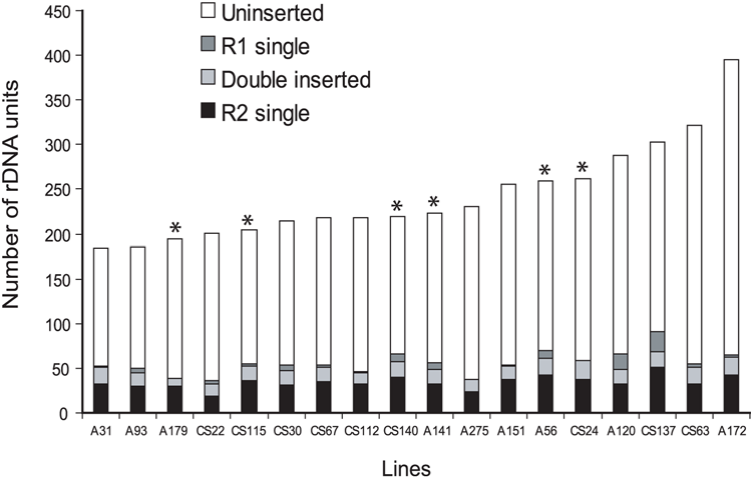
Composition of the rDNA loci of 18 iso-rDNA lines was determined by genomic blot analysis. Bar heights indicate the total number of rDNA units in each line. The shading of the bar indicates the number of units that are uninserted or inserted with R1, R2 or both elements. Asterisks indicate the six lines with the highest levels of R2 transcripts.

The total number of rDNA units (locus size) in each of the 18 lines were estimated by dividing the number of R2 elements by the fraction of the rDNA units inserted with R2 (Figure 7). The rDNA loci varied in size from 185 units to 395 units. Shown in Figure 6C is a plot of rDNA locus size versus the R2 transcript levels. While two of the lines with the lowest levels of R2 transcript did have the two largest rDNA loci, there was no significant correlation between rDNA locus size and R2 transcript level across all 18 lines (r = −0.02, p = 0.48).

These findings suggest that unlike our study of laboratory stocks, there was no increase in the number of R2 elements in those rDNA lines with high levels of R2 transcripts. The only significant correlation was a small decrease in the fraction of inserted rDNA units in those lines with essentially no R2 transcripts. Finally, it should be noted that R1-inserted units were at low levels in all stocks (Table 1) and their estimated numbers or fraction of the total rDNA units inserted did not correlate with R2 transcript levels (data not shown).

### R2 Distribution in the rDNA Locus

**Figure 8:**
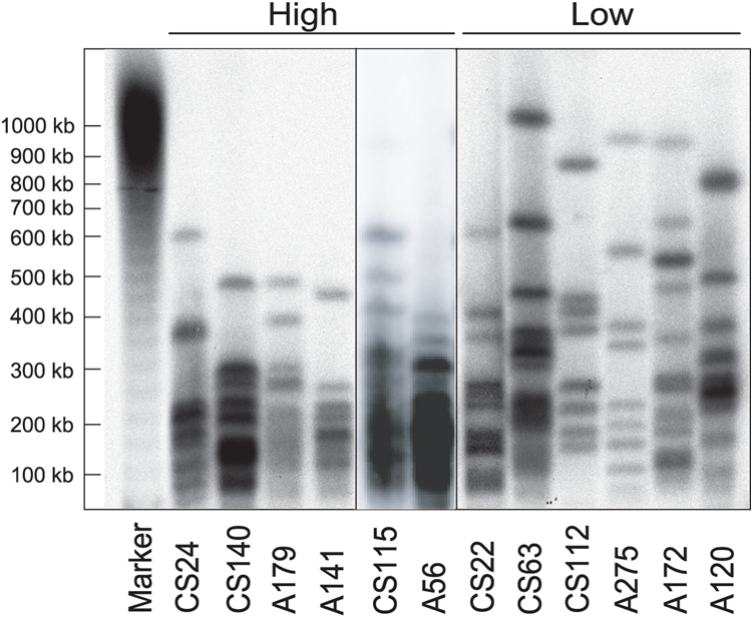
Distribution of R2 elements in the rDNA locus. High molecular weight DNA was prepared from 0–23 hr old embryo nuclei, digested with *Not*I, which cleaves only in the R2 element (see Figure 1), and separated by pulsed-field electrophoresis. Six lines with high levels of R2 transcripts (High) and six lines with little or no R2 transcripts (Low) were used in the analysis. After transfer to nitrocellulose the DNA was probed with a segment from the 18S rRNA gene [26]. The DNA marker standard corresponds to a 50 kb ladder. Two samples (CS115 and A56) were analyzed on a separate gel under identical conditions. Also because of a lower DNA concentration in the nuclear plug, the CS22 lane represents a longer exposure of the DNA blot.

Because neither the size nor the composition of the rDNA locus exhibited a dramatic correlation with the level of R2 transcripts, the distribution of R2 insertions within the locus was next determined. The restriction enzyme *Not*I cleaves near the middle of the R2 element (Figure 1), but does not cleave R1 elements or the transcribed and intergenic spacer regions of the *D. simulans* rDNA units. Thus the size of genomic *Not*I fragments detected in a line reveals the R2-to-R2 spacing within its rDNA locus; however, 3–12 highly 5′ truncated R2 copies in each line (see Figure 4) will not be mapped by this approach. To determine the distribution of R2 elements in the rDNA loci of the low and high transcript lines, nuclei were isolated from 0–23 hr embryos, embedded in agarose, gently lysed and the DNA digested with *Not*I [26]. The DNA fragments were separated by pulsed-field electrophoresis, transferred to nitrocellulose and hybridized with a DNA fragment from the 18S rRNA gene. The six lines with the highest levels of R2 transcription as well as six lines with the lowest levels of R2 transcripts were used for this study (Figure 8).

The pulse times for electrophoresis were established to optimize the separation of the largest *Not*I fragments generated from the rDNA loci. Under these conditions *Not*I fragments below 100 kb are not well separated from the buffer front moving through the gel. Thus those portions of the rDNA locus containing R2 elements separated by less than eight rDNA units (the average rDNA unit size is about 12 kb) are not present in the figure. These closely spaced R2-inserted units represent a small fraction of the rDNA locus (∼25%) but a large fraction of the R2 elements (∼75%).

The *D. simulans* lines with highest levels of R2 transcripts had on average smaller *Not*I fragments than the lines with low levels of R2 transcripts. This difference in size was significant, whether one used only the largest *Not*I fragment (∼800 kb for the low lines and 540 kb for the high lines, Mann-Whitney U test, p = 0.003), or the combined length of the three largest fragments in each line (∼1850 kb for the low lines and 1200 kb for the high lines, p = 0.004). This finding suggests that of the various properties of the rDNA locus that were measured, the distribution of R2 elements within the locus appeared to best serve as an indicator of whether R2-inserted units were transcribed. However, even this difference in the spacing of R2-inserted units is subtle in that changes in the location of only a small number of R2 elements within the locus could shift the profile from the low to high transcript patterns.

## Discussion

The goal of this study was to apply what had been learned about R2 activity from our studies of laboratory stocks to characterize the pattern of R2 activity in natural populations of *D. simulans*. In our previous analyses nuclear run-on transcription experiments indicated that control over R2 element activity was at the level of transcription, while crosses between active and inactive lines revealed that this transcriptional control mapped to the site of all R2 insertions, the rDNA locus on the X chromosome [26]. Therefore, in this study iso-rDNA locus lines were established from two natural populations and their levels of R2 transcripts determined within a few generations of isolation. In both populations, about one-half of the lines had levels of R2 transcripts that were very low to undetectable. In the remaining lines, the level of R2 transcript varied in a continuous manner over a wide range. In the 18 lines studied as representative of the R2 transcript range, a positive correlation was detected between R2 transcript level and R2 retrotransposition activity. While this correlation was not absolute, transcript level appeared to serve as a reliable indicator of potential R2 activity and thus a major means of control over R2 activity in a population. Structural analysis of the rDNA loci of the 18 lines indicated that the increased R2 transcription was not a result of the greater accumulation of R2 copies or changes in the size of the rDNA locus.

The property of the rDNA locus that best correlated with the level of R2 transcripts was the size of the largest continuous stretch of units free of R2 insertions. This suggestion that it is a difference in the distribution of R2 elements within the rDNA locus that controls R2 transcription adds considerable support to our previously proposed model of R2 regulation [26]. This model was based on several known properties concerning the transcription of rDNA units and their R2 insertions [26]. First, R2 elements do not appear to encode their own promoter; rather their RNA transcripts are processed from a co-transcript with the 28S rRNA gene [26], [32]–[35]. Second, the size of the rDNA locus is sufficiently large that only a fraction of the rDNA units encoded by an organism are transcribed at any one time [23], [36]–[39]. Third, electron microscopic observations suggest that transcriptionally active rDNA units occur in contiguous blocks rather than as dispersed units throughout the locus [40],[41]. Based on these observations we proposed that arthropods have evolved the ability to identify and actively transcribe one or more regions of the rDNA locus that contain the lowest frequency of R2-inserted units [26]. Thus significant transcription of R2 elements occurs when an organism is unable to identify large continuous regions of the rDNA locus free of insertions.

In the laboratory stocks used in our previous study, there was on average a two-fold greater number of R2 elements in active lines compared to the inactive lines. As a consequence it was not possible to exclude the model that the greater number of R2 insertions in the rDNA loci led to higher transcription levels. In our natural lines, on the other hand, the number of R2 insertions was similar between the active and inactive lines, suggesting that it is R2 distribution, not number, that determines whether R2-inserted units are transcribed. Our study also suggests that it may be the location of only a few R2 elements that determines the level of R2 activity within a line. A repositioning of only a small number of R2 elements could change the *Not*I digestion pattern of the low transcript lines to that of the high transcript lines (Figure 8). In addition, our determination of transcript level was based on the level of full-length R2 transcripts, while the R2 distribution pattern was based on the presence of a *Not*I site near the middle of the R2 element. Thus the positioning of 5′ truncated R2 copies, that are of a length to retain the *Not*I site but not able to generate full-length transcripts, within the regions of lowest R2 abundance could explain why some lines low transcript lines (e.g. CS22) have *Not*I profiles more similar to a high transcript line.

Our analysis of the individual R2 elements present in the rDNA loci of each line (Figure 4) revealed that each rDNA locus had different collections of R2 insertions suggesting that R2 elements are continually being gained and lost within a population. The rDNA loci should, therefore, be viewed as a continuously changing landscape with the individual loci shifting between low and high levels of R2 transcription. These continuously changing rDNA loci can explain another difference between natural and laboratory stocks. Of the 15 lines isolated from Paradise, CA and maintained in the laboratory for about 10 years, about one-fourth (4 lines) had extremely high levels of R2 transcripts and retrotransposition [24],[26]. However, of the 180 lines established from the Atlanta and San Diego populations, only 2% had transcript levels as high as that seen in the laboratory stocks, and only one of these lines had levels of retrotransposition as high as the laboratory stocks. While it is formerly possible that the Paradise population simply had higher levels of R2 activity, it appears more likely that during the prolonged period the Paradise stocks were maintained in the laboratory the inbreeding and relaxed selection conditions permitted the accumulation of the higher levels of R2 elements observed in these active laboratory stocks. Such shifts to high R2 element activity may also occur in the natural populations from Atlanta and San Diego, but such flies would be lost from the populations by natural selection. We have found that laboratory stocks with high levels of R2 activity are readily out-competed when crossed to laboratory stocks containing low levels of R2 activity (D. Eickbush, unpublished data).

One important feature of R2 control detected in our previous study of laboratory stocks was the dominance of expression of rDNA loci with low levels of R2 transcripts over loci with high levels of R2 transcripts [26]. In females containing one rDNA locus from a stock with high levels of R2 transcription (R2 active locus) and one locus from a stock with no R2 transcription (R2 inactive locus), rDNA units in the R2 inactive loci were preferentially transcribed. We termed this ability to turn off an entire rDNA locus nucleolar dominance, as it appeared similar to the frequent dominance of one rDNA locus in interspecies hybrids [42]. To determine if such dominance could also be detected between our natural rDNA loci, we conducted reciprocal crosses between the most active line in this study, CS24, to several inactive lines. In the heterozygous female offspring of both crosses the high level of R2 transcript seen from the CS24 loci was significantly turned off, consistent with the dominance of R2 inactive loci in natural populations (data not shown). However, such crosses involving lines with the more typical lower levels of transcripts were often not recessive to R2 inactive stocks. Thus, while more experiments are needed, nucleolar dominance may only influence loci with extremely high levels of transcripts.

Based on our survey of the two *D. simulans* populations we can estimate an R2 retrotransposition rate for the entire population. Comparing the rate of R2 retrotransposition with the level of transcripts (Figure 5), about 50% of the rDNA loci in the two populations are likely to undergo no retrotransposition. The remaining rDNA loci have a high probability of supporting some level of retrotransposition. For the 12 rDNA loci we sampled from this group, 46 retrotranspositions were detected when 16 chromosomes were monitored in the eighth generation giving a rate of 0.034 events/chromosome/generation [46 events / (12 lines ×7 generations ×16 chromosomes)]. As a lower limit, if we exclude the one extremely active line from this analysis the R2 retrotransposition rate drops to 0.019 events/chromosome/generation. Because only 5′ truncated R2 elements, which represent about half the total number of elements, were monitored in our assays, these rates should be multiplied by a factor of two (range of 0.038–0.068). Finally, correcting for the fraction of the rDNA loci that have detectable levels of transcription (50%) the total R2 retrotransposition rate for the population can be estimated at 0.019–0.034 events/chromosome/generation. While this range is only a rough estimate, it is consistent with computer simulations conducted to mimic the types of recombination and rates of retrotransposition likely to be present in the rDNA loci of *Drosophila* [23].

The findings in this report address one of the major questions concerning the ability of transposable elements to maintain themselves within a species lineage. Does the control mechanism of the cell occasionally breakdown, or is this mechanism simply incapable of preventing all new insertions? In the case of R2 our findings support the latter possibility. In most organisms, large numbers of R2 elements can be effectively prevented from transcription by expressing the rDNA units in only a small domain that does not contain R2 insertions. However, recombination events (in particular unequal crossovers) will expand, contract and rearrange the rDNA units in the locus meaning these domains free of R2 insertion are not permanently stable. Thus it is not a difference in the cellular control mechanism between animals in a population, but rather differences in their rDNA loci that determines whether R2 elements are being transcribed. The long-term stability of R2 elements is assured because even though all copies can be effectively silenced for long periods of time, the transcription of some copies will eventually be resurrected by recombination.

Of course transcriptional control may not be the only level at which the activity of R2 elements are regulated. While retrotranspositions have never been detected in a stock with no detectable R2 transcripts, there may not be a complete correlation between the level of R2 transcripts and the rate of retrotransposition. Retrotransposition events were difficult to detect in one of the lines with the highest level of R2 transcripts (Figure 5). In one of our laboratory stocks, retrotransposition events were more frequent than expected based on R2 transcript levels [26]. Thus additional post-transcriptional steps appear to influence the rate of R2 retrotransposition in *D. simulans*. Further studies are needed to determine if these steps represents significant control mechanisms over R2 activity.

Finally, this population study not only provides additional support for a domain model of transcription in the rDNA locus but also addresses two evolutionary questions raised by our earlier studies. First, why do the different copies of R2 generated by retrotransposition usually remain single-copy (i.e. are not duplicated by recombination) until they are eventually lost from the genome [24],[27],[35]? Second, why in long-term studies of specific rDNA loci did the number of uninserted rDNA units change most rapidly than the inserted units [29]? Both of these finding can be explained if recombination events in the rDNA loci predominantly occur in the insertion free region of the rDNA locus activated for transcription. Thus an understanding of R2 location and control is needed to appreciate the forces at work in the expression and evolution of the entire rDNA locus.

## Materials and Methods

### Fly Sources

Isofemale stocks of *D. simulans* collected in San Diego, CA were generated by Peter Andolfatto. *D. simulans* females of Atlanta population were collected by Todd Schlenke in Atlanta, GA. The Beadex (Bx) marker line was obtained from Allen Orr.

### Establishment of Iso-rDNA Lines

The single rDNA locus of *D. simulans* is located on the X chromosome. The rDNA locus of the isolated stocks was followed using Bx, a dominant wing phenotype marker that is the closest available marker to the rDNA locus in *D. simulans*. First (Atlanta) or second generation (San Diego) male progeny of collected females were crossed to Bx females. F1 females from these crosses were then backcrossed to the original male. F2 females homozygous for the wild rDNA loci (X^w^X^w^) were crossed to X^w^Y F2 males to generate the individual lines. Because heterozygous Bx females (X^Bx^X^w^) do not always express the Bx wing phenotype, three single pair crosses between an F2 female and an F2 male were performed. Only if all F3 males from a pair showed the normal wing phenotype was that line considered to contain only the wild-type rDNA locus (iso-rDNA lines). One iso-rDNA locus line was derived from each female collected from the natural population. Because recombination at a low level can occur between Bx and the rDNA locus, the origin of the rDNA loci was confirmed in those lines used for more extensive studies by comparing the R2 5′ truncation profiles of multiple males with the R2 5′ truncation profiles of the original male and the Bx stock.

### PCR Amplification to Score R2 5′ Truncation Profiles

Genomic DNA was extracted from single flies as described by Gloor et al. [43]. To compare the R2 elements between lines as well as count the total number of R2 elements in each line, the 5′ ends of all R2 insertions were analyzed by PCR amplification. The forward primer, 5′-TGCCCAGTGCTCTGAATGTC-3′, which annealed to 28S gene sequences 80 bp upstream of the R2 insertion site, was ^32^P-5′-end-labeled and used with the following series of reverse primers that annealed to R2 sequences at various distances from the 5′ end of a full-length element: 3.6 kb (5′-GTATGGAAATCTATCGAAAGATACT-3′), 3.1 kb (5′-GTCACCTGCGGCTTCGAATC-3′), 2.8 kb (5′- CCCCTTGTAGTACGAGACTTC-3′), 2.6 kb (5′-GCCGGACGCGATAACAATTC-3′), 2.0 kb (5′-GATAGAAAATCCAACGTTCTGTC-3′), 1.6 kb (5′-TCGAATGCCTTGCTTACATC-3′), 1.3 kb (5′-GAAGACGGTTCTGGCCAGTC -3′), 1.0 kb (5′-CGCTGGACGACAGCATACTGC-3′), 0.4 kb (5′-CATCAAGTTCGTCTGGGTGC-3′) and 0.1 kb (5′-GACTTGAGTAAAGGAGAGACT-3′). The labeled PCR products were separated on an 8% high voltage denaturing polyacrylamide gel, exposed to a PhorsphorImager screen, and quantitated with a Molecular Dynamics PhorsphorImager scanner using ImageQuant software. Most PCR bands (est. 80%) were of equal intensity and corresponded to single copies of the R2 element. To quantify the bands derived from multiple copies of R2 with identical length 5′ ends, the intensity of each PCR band was calculated with a regression analysis using the many single copy variants as reference [29]. While labor intensive, we have found this approach to be more accurate than quantitative PCR or relative hybridizations [24],[27],[29],[35]. To score R2 retrotransposition and deletion events, the upstream (forward) primer without end-labeling was used with the above set of reverse PCR primers, separated on 8% native polyacrylamide gels and stained with ethidium bromide.

### RNA Blots to Detect R2 Transcript

Total RNA was extracted as previously described [35] from 30 adult females of each line in the third generation. Ten micrograms of RNA were separated on 1% agarose, 2.2 M formaldehyde gels, the RNA transferred to GeneScreen Plus, and hybridized with an anti-sense RNA probe from the 5′ end of the R2 element (probe 1, Figure 1) as previously described [26]. The relative levels of 3,600 nt full-length R2 transcripts were quantitated on a PhorsphorImager. As a control for RNA loading and quality, all R2 hybridization signals were standardized by monitoring the level of alcohol dehydrogenase hybridization on the same blots [26]. As a control for hybridization efficiency, an equal aliquot of RNA isolated from the previously characterized lab stock, line 58 [24] was included in each blot.

### Genomic DNA Blots to Determine R2 Insertion Level and rDNA Locus Size

Genomic DNA was extracted from 30–40 adult females from each line in the fifth generation. To determine the proportions of uninserted, R1-inserted and R2-inserted rDNA units, the genomic DNA was digested with *Cla*I and *Pst*I, fractionated through a 1% agarose gel, and transferred to nitrocellulose. The blot was probed with a segment of the 28S gene (probe 2, Figure 1) located downstream of the R2 insertion site [29]. Uninserted units gave rise to a 2.3 kb *Cla*I-*Cla*I fragment, R1-inserted and doubly inserted units to a 1.5 kb *Pst*I-*Cla*I fragment, and R2-inserted units to a 1.3 kb *Pst*I-*Cla*I fragment. To determine the proportions of R1 and R2 double-inserted units, another aliquot of genomic DNA was digested with *MspA1*I and hybridized with a fragment from the 3′ end of R2 (probe 3, Figure 1). The sequence and location of this probe were described by Perez-Gonzalez and Eickbush [10]. Because R2 insertions are located a short distance upstream of R1 insertions, R2-single inserted units gave rise to a 2.2 kb hybridizing fragment, while R2 / R1-double inserted units gave rise to a 1.3 kb fragment. The size of the rDNA locus was estimated as the number of R2 elements divided by the fraction of the rDNA locus containing R2 insertions. All data on the fraction of rDNA units of each insertion class represents the mean and standard error obtained from four separate blots.

### Pulsed-Field Gel Electrophoresis

Nuclei isolation from 0–23 hour embryos, suspension in 1% InCert agarose, DNA purification, and *Not*I restriction digestion were conducted as previously described [26]. The digested nuclei plugs were subjected to pulsed-field electrophoresis in 1% agarose at 12°C on a CHEF-DRII apparatus (Promega) for 30 hours at 175 volts with switch times of 10 to 30 seconds. The DNA transfer to nitrocellulose and the hybridization a fragment of the 18S rRNA gene were conducted as previously described [26].

## References

[1] Charlesworth B, Langley CH (1989). The population genetics of Drosophila transposable elements.. Annu Rev Genet.

[2] Charlesworth B, Sniegowski P, Stephan W (1994). The evolutionary dynamics of repetitive DNA in eukaryotes.. Nature.

[3] Slotkin RK, Martienssen R (2007). Transposable elements and the epigenetic regulation of the genome.. Nat Rev Genet.

[4] Vastenhouw NL, Plasterk RH (2004). RNAi protects the *Caenorhabditis elegans* germline against transposition.. Trends Genet.

[5] Finnegan DJ (1992). Transposable elements.. Curr Opin Genet Dev.

[6] Biemont C, Lemeunier F, Gautier C, Garcia Guerreiro M, Aulard S (1994). High rate of movement of one (mdg3) out of four transposable elements in a natural population of *Drosophila melanogaster*.. C R Acad Sci III.

[7] Nuzhdin SV, Mackay TF (1995). The genomic rate of transposable element movement in *Drosophila melanogaster*.. Mol Biol Evol.

[8] Prud'homme N, Gans M, Masson M, Terzian C, Bucheton A (1995). Flamenco, a gene controlling the *gypsy* retrovirus of *Drosophila melanogaster*.. Genetics.

[9] Vieira C, Biemont C (1997). Transposition rate of the 412 retrotransposable element is independent of copy number in natural populations of *Drosophila simulans*.. Mol Biol Evol.

[10] Perez-Gonzalez CE, Eickbush TH (2001). Dynamics of R1 and R2 elements in the rDNA locus of *Drosophila simulans*.. Genetics.

[11] Desset S, Meignin C, Dastugue B, Vaury C (2003). COM, a heterochromatic locus governing the control of independent endogenous retroviruses from *Drosophila melanogaster*.. Genetics.

[12] Eickbush TH, Eickbush DG (2007). Finely orchestrated movements: evolution of the ribosomal RNA genes.. Genetics.

[13] Ganley AR, Kobayashi T (2007). Highly efficient concerted evolution in the ribosomal DNA repeats: total rDNA repeat variation revealed by whole-genome shotgun sequence data.. Genome Res.

[14] Stage DE, Eickbush TH (2007). Sequence variation within the rRNA gene loci of 12 Drosophila species.. Genome Res.

[15] Eickbush TH, Craig N, Craige R, Gellert M, Lambowitz A (2002). R2 and related site-specific non-long terminal repeat retrotransposons.. Mobile DNA II.

[16] Penton EH, Crease TJ (2004). Evolution of the transposable element Pokey in the ribosomal DNA of species in the subgenus *Daphnia* (*Crustacea: Cladocera*).. Mol Biol Evol.

[17] Kojima KK, Fujiwara H (2005). Long-term inheritance of the 28S rDNA-specific retrotransposon R2.. Mol Biol Evol.

[18] Burke WD, Malik HS, Lathe WC, Eickbush TH (1998). Are retrotransposons long-term hitchhikers?. Nature.

[19] Malik HS, Burke WD, Eickbush TH (1999). The age and evolution of non-LTR retrotransposable elements.. Mol Biol Evol.

[20] Jakubczak JL, Zenni MK, Woodruff RC, Eickbush TH (1992). Turnover of R1 (type I) and R2 (type II) retrotransposable elements in the ribosomal DNA of *Drosophila melanogaster*.. Genetics.

[21] Lathe WD, Eickbush TH (1997). A single lineage of r2 retrotransposable elements is an active, evolutionarily stable component of the Drosophila rDNA locus.. Mol Biol Evol.

[22] Lathe WC, Burke WD, Eickbush DG, Eickbush TH (1995). Evolutionary stability of the R1 retrotransposable element in the genus Drosophila.. Mol Biol Evol.

[23] Zhang X, Eickbush MT, Eickbush TH (2008). Role of recombination in the long-term retention of transposable elements in rRNA gene loci.. Genetics.

[24] Zhang X, Eickbush TH (2005). Characterization of active R2 retrotransposition in the rDNA locus of *Drosophila simulans*.. Genetics.

[25] Zhang X, Zhou J, Eickbush TH (2008). Rapid R2 retrotransposition leads to the loss of previously inserted copies via large deletions of the rDNA locus.. Mol Biol Evol.

[26] Eickbush DG, Ye J, Zhang X, Burke WD, Eickbush TH (2008). Epigenetic Regulation of Retrotransposons within the Nucleolus of Drosophila.. Mol Cell Biol.

[27] Perez-Gonzalez CE, Eickbush TH (2002). Rates of R1 and R2 retrotransposition and elimination from the rDNA locus of *Drosophila melanogaster*.. Genetics.

[28] George JA, Burke WD, Eickbush TH (1996). Analysis of the 5′ junctions of R2 insertions with the 28S gene: implications for non-LTR retrotransposition.. Genetics.

[29] Averbeck KT, Eickbush TH (2005). Monitoring the mode and tempo of concerted evolution in the *Drosophila melanogaster* rDNA locus.. Genetics.

[30] Burke WD, Eickbush DG, Xiong Y, Jakubczak J, Eickbush TH (1993). Sequence relationship of retrotransposable elements R1 and R2 within and between divergent insect species.. Mol Biol Evol.

[31] Eickbush DG, Eickbush TH (1995). Vertical transmission of the retrotransposable elements R1 and R2 during the evolution of the *Drosophila melanogaster* species subgroup.. Genetics.

[32] Long EO, Dawid IB (1979). Expression of ribosomal DNA insertions in of *Drosophila melanogaster*.. Cell.

[33] Kidd SJ, Clover DM (1981). *Drosophila melanogaster* ribosomal DNA containing type II insertions is variably transcribed in different strains and tissues.. J Mol Biol.

[34] George JA, Eickbush TH (1999). Conserved features at the 5 end of *Drosophila* R2 retrotransposable elements: implications for transcription and translation.. Insect Mol Biol.

[35] Eickbush DG, Eickbush TH (2003). Transcription of endogenous and exogenous R2 elements in the rRNA gene locus of *Drosophila melanogaster*.. Mol Cell Biol.

[36] Ye J, Eickbush TH (2006). Chromatin structure and transcription of the R1- and R2-inserted rRNA genes of *Drosophila melanogaster*.. Mol Cell Biol.

[37] Dammann R, Lucchini R, Koller T, Sogo JM (1995). Transcription in the yeast rRNA gene locus: distribution of the active gene copies and chromatin structure of their flanking regulatory sequences.. Mol Cell Biol.

[38] Conconi A, Widmer RM, Koller T, Sogo JM (1989). Two different chromatin structures coexist in ribosomal RNA genes throughout the cell cycle.. Cell.

[39] McKnight SL, Miller OL (1976). Ultrastructural patterns of RNA synthesis during early embryogenesis of *Drosophila melanogaster*.. Cell.

[40] Chooi WY (1979). The occurrence of long transcription units among the X and Y ribosomal genes of *Drosophila melanogaster*: transcription of insertion sequences.. Chromosoma.

[41] Jamrich M, Miller OL (1984). The rare transcripts of interrupted rRNA genes in *Drosophila melanogaster* are processed or degraded during synthesis.. EMBO J.

[42] Pikaard CS (2000). Nucleolar dominance: uniparental gene silencing on a multi-megabase scale in genetic hybrids.. Plant Mol Biol.

[43] Gloor GB, Preston CR, Jonhnson-Schlitz DM, Nassif NA, Phillis RW (1993). Type I repressors of *P*-element mobility.. Genetics.

